# Biogenic Volatile Organic Compound and Respiratory CO_2_ Emissions after ^13^C-Labeling: Online Tracing of C Translocation Dynamics in Poplar Plants

**DOI:** 10.1371/journal.pone.0017393

**Published:** 2011-02-28

**Authors:** Andrea Ghirardo, Jessica Gutknecht, Ina Zimmer, Nicolas Brüggemann, Jörg-Peter Schnitzler

**Affiliations:** Karlsruhe Institute of Technology, Institute for Meteorology and Climate Research, Garmisch-Partenkirchen, Germany; University of Sheffield, United Kingdom

## Abstract

**Background:**

Globally plants are the primary sink of atmospheric CO_2_, but are also the major contributor of a large spectrum of atmospheric reactive hydrocarbons such as terpenes (e.g. isoprene) and other biogenic volatile organic compounds (BVOC). The prediction of plant carbon (C) uptake and atmospheric oxidation capacity are crucial to define the trajectory and consequences of global environmental changes. To achieve this, the biosynthesis of BVOC and the dynamics of C allocation and translocation in both plants and ecosystems are important.

**Methodology:**

We combined tunable diode laser absorption spectrometry (TDLAS) and proton transfer reaction mass spectrometry (PTR-MS) for studying isoprene biosynthesis and following C fluxes within grey poplar (*Populus* x *canescens*) saplings. This was achieved by feeding either ^13^CO_2_ to leaves or ^13^C-glucose to shoots via xylem uptake. The translocation of ^13^CO_2_ from the source to other plant parts could be traced by ^13^C-labeled isoprene and respiratory ^13^CO_2_ emission.

**Principal Finding:**

In intact plants, assimilated ^13^CO_2_ was rapidly translocated via the phloem to the roots within 1 hour, with an average phloem transport velocity of 20.3±2.5 cm h^−1^. ^13^C label was stored in the roots and partially reallocated to the plants' apical part one day after labeling, particularly in the absence of photosynthesis. The daily C loss as BVOC ranged between 1.6% in mature leaves and 7.0% in young leaves. Non-isoprene BVOC accounted under light conditions for half of the BVOC C loss in young leaves and one-third in mature leaves. The C loss as isoprene originated mainly (76–78%) from recently fixed CO_2_, to a minor extent from xylem-transported sugars (7–11%) and from photosynthetic intermediates with slower turnover rates (8–11%).

**Conclusion:**

We quantified the plants' C loss as respiratory CO_2_ and BVOC emissions, allowing in tandem with metabolic analysis to deepen our understanding of ecosystem C flux.

## Introduction

Plant CO_2_ assimilation is a fundamental sink for atmospheric CO_2_ in the global carbon (C) cycle, amounting to a gross CO_2_ uptake of approximately 120 Gt C per year by terrestrial plants [1]. This amounts to 15–20 times more than is currently emitted as CO_2_ in the course of anthropogenic activities [2]. A substantial part of the photosynthetically fixed C is not stored in plant tissues, but re-emitted to the atmosphere as a wide range of volatile C substances, including respired CO_2_ and biogenic volatile organic compounds (BVOC, e.g., isoprene). In addition, roots excrete labile C compounds, which can be used by microorganisms and in turn be re-emitted back to the atmosphere, mainly as CO_2_ or methane. Due to the reactive nature, plant BVOC have a significant impact on atmospheric chemistry. The influence of plant BVOC on atmospheric chemistry includes the formation of tropospheric ozone, carbon monoxide and hydroxyl radical levels, methane half-life, and secondary aerosols [3], [4], [5], [6]. Thus, due to their importance for air quality and climate dynamics there is much interest to understand their biosynthesis and regulation [7].

Part of the assimilated C is translocated from net sources (e.g., mature leaves) to net sinks (e.g., roots, apical buds, growing tissues) in order to sustain plant development, growth and metabolism in both green and non-green tissues. Understanding how these allocation and translocation patterns are affected by environmental constraints is crucial for predicting the future C uptake capacity of terrestrial ecosystems.

In poplar, 2–5% of the assimilated C is instantaneously re-emitted to the atmosphere as isoprene [8]. Although isoprene is the most important BVOC emitted by poplar, other BVOC (e.g., aldehydes, alcohols, mono- and sesquiterpenes) might contribute significantly to the overall C loss of poplars, but are often ignored due to technical limitations of VOC analysis.

Light-dependent emission of isoprene is closely related to photosynthesis, which provides the required C precursors and energy and redox equivalents for the plastidic isoprenoid pathway (e.g.: [9], [10]). Under unstressed conditions, atmospheric CO_2_ is the main (approximately 80%) C source of isoprene formation. Other “old” C, such as xylem sap sugars, starch and re-fixation of CO_2_ originating from mitochondrial metabolism [9], [11], [12], [13] act as “alternative” C source [13] and therefore contribute significantly to isoprene biosynthesis. When photosynthesis is impaired by environmental stresses (e.g., drought, salinity), isoprene biosynthesis becomes transiently uncoupled from direct atmospheric CO_2_ uptake [7]. Under these circumstances, C previously allocated to storage reservoirs (starch) or transported soluble C might sustain isoprene formation, supplying the plastidic isoprenoid pathway during impaired photosynthetic activity [14].

Up to now, information on the contribution of C fixed within a leaf, relative to C fixed in other leaves, on isoprene formation is missing. There is evidence that C translocation within the plant is an immediate process following the fixation of atmospheric CO_2_. Photosynthetic activity affects soil-respired CO_2_ within a few hours in non-woody plants such as grasses [15], and within a few days (1–5 d) in mature trees [16], [17], [18], [19], [20]. Studies on pedunculate oak (*Quercus robur* L.) [21] and grey poplar (*Populus canescens* (Aiton) Sm.) [22] saplings demonstrated that a significant amount of C is translocated via the transpiration stream, particularly at enhanced carbon demand or reduced photosynthesis. There is indication that xylem-transported C can serve as an alternative C source for isoprene biosynthesis as showed previously by feeding single detached poplar leaves with ^13^C-glucose via the petiole [13]. However, no information is available how this xylem source truly contributes to isoprene biosynthesis in plant, where leaves have not been detached from their stem. Also, it has not been investigated whether source or sink of photosynthates (i.e. mature leaves against young and developing leaves) equally benefit from this additional C source or not.

Here we perform a ^13^C-labeling experiment on poplar plants and we aim to: i) monitor online plant C fluxes as markers of C translocation processes; ii) determinate C allocation in different plant parts; iii) test the hypothesis that recently translocated C might be immediately used as “alternative” C sources for isoprene and its precursor dimethylallyl pyrophosphate (DMADP) biosynthesis; iv) quantify the contribution of xylem-derived sugars; and v) understand the xylem-mediated source of isoprene formation; vi) obtain a comprehensive picture of most of the volatile organic compounds emitted from poplar trees.

In order to achieve our goals, we used a new non-destructive ^13^C-labeling approach by combining a tunable diode laser absorption spectrometer (TDLAS) and a proton transfer reaction mass spectrometry (PTR-MS) to measure respiratory CO_2_ and BVOC emission from different plant parts in real time. To achieve this, the roots, mature leaves, young leaves and the apical part were enclosed in a system of four parallel cuvettes [10]. We applied the combination of both techniques for tracing ^13^C fluxes in real time from the site of ^13^CO_2_ fixation in a mature poplar leaf, into isoprene and respiratory CO_2_ emitted from different plant parts. In addition we labeled cut shoots in parallel by fumigating leaves with ^13^CO_2_ or feeding the shoots with ^13^C-glucose via xylem uptake.

Thus, the incorporation of ^13^C into respiratory CO_2_ was used as an isotopic marker of the C translocation process. The effects of exposure to ^13^C-labeling were investigated on the level of isoprene biosynthesis. Finally, in order to investigate the effects on isoprene biosynthesis in absence of its main C source, we deprived plant parts of atmospheric CO_2_. Under this unnatural stress condition, we followed the alteration of C fluxes, CO_2_ respiration, and BVOC emissions.

## Materials and Methods

### Plant material and growth conditions

All experiments were performed with one-year-old grey poplar saplings (hybrid of *Populus tremula* x *P. alba*, syn. *Populus* x *canescens* (Aiton) Sm.). Cultivation procedure and growing conditions are described elsewhere [23], [24].

### Experimental design

**Figure 1:**
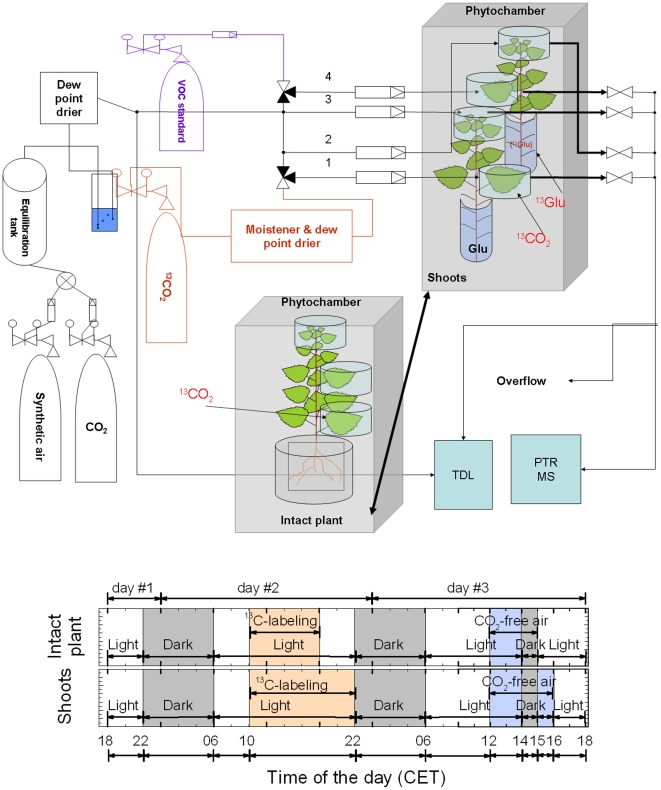
Scheme of the experimental design. Ten liter per minute of synthetic VOC-free air (BASI Schöberl, Germany) were mixed with CO_2_ (38,000 ppmv) to a final concentration (in the cuvette) of 385 ppmv, passing a 20 L equilibration tank before being completely humidified by bubbling the airstream through pure, distilled water. A dew point unit assured a stable humidity level before the airflow entered each of the four cuvettes through flow controllers, set at 2 L min^−1^. One cuvette (# 1) could be connected via a 3-port valve to a separate ^13^CO_2_-tank for ^13^C-labeling. The gas was purchased already mixed at 385 ppmv ^13^CO_2_ and humidified separately using a portable dew point generator (Li-610, Licor, Lincoln, NE, USA). Another cuvette (# 4) was connected to a VOC standard mixture for the calibration of the PTR-MS. Inlet and outlet of the cuvettes were directed sequentially via electronic, computer controlled 3-port valves to the three gas analyzers (LI-7000, Licor; or GFS-3000, Heinz Walz, Germany; TDLAS; PTR-MS), while the excess flow was directed to a vent which was periodically checked for flow rate in order to ensure that the whole system was gas-tight. The grey boxes display the two different experimental designs: either with intact plant placed into hydroponic solution where one single mature leaf was labeled with ^13^CO_2_ or with two parallel shoots labeled either with ^13^CO_2_ or ^13^Glc.

The general schematic overview of the experimental setup is illustrated in Fig. 1. Experiments were conducted on either i) intact plants in hydroponic culture, or ii) shoots without a root system. Hydroponic cultures were established 7–10 d prior to the start of the experiments. Soil was carefully removed from the roots, which were planted in 100% perlite substrate with sterile Long Ashton nutrient solution [25]. For experiments with shoots, the root systems were cut off the same morning of the experiment. In order to avoid embolism during cutting, plant transpiration was lowered by keeping the plants for 2–4 h in a cold room in the dark. Branches were then cut under water and transferred to 50-mL flasks containing autoclaved Long Ashton nutrient solution with 10 mM unlabeled glucose (^12^Glc). The poplar stems were inserted into the glucose-containing flasks, which were sealed with parafilm, by punching through the parafilm.

All experiments were performed using dynamic leaf cuvette systems made of aluminum and perspex glass [10]. For CO_2_ released from roots, the hydroponic culture was enclosed by a bigger perspex cuvette (volume  = 5670 cm^3^) than the pot containing the hydroponic culture (volume  = 2000 cm^3^). Leaves, apical bud and the root system were placed in the cuvettes the day before ^13^C-labeling. Inside each cuvette, leaf temperature was measured with a thermocouple touching the bottom of the enclosed leaf, and maintained at 30°C with computer-controlled Peltier elements. Light at 1000 µmol m^−2^ s^−1^ PPFD was supplied by an LED array on top of each cuvette during light phase measurements (06:00–22:00 CET). Experiments were conducted inside a phytochamber. The entire phytochamber was kept at the same conditions as prevailing inside the cuvettes.

The ^13^C label was applied in two different ways: either (i) ^13^CO_2_ was fed to a fully mature source leaf of intact plants or cut shoots by replacing CO_2_ with natural ^13^C abundance with ^13^CO_2_ (99 atom% ^13^C; Air Liquide, Krefeld, Germany) at the same concentration (385 ppmv), or (ii) ^13^C-glucose (^13^Glc) was supplied to the transpiration stream via the xylem of cut shoots by replacing a Glc solution containing ^13^C at natural abundance (10 mM) with an equimolar universal labeled ^13^Glc solution (99 atom% ^13^C; Cambridge Isotope Laboratories, Andover, MA, USA).

In experiments with intact plants, the four cuvettes were run in parallel on a single plant to detect exchange of ^13^C between mature leaves, the apex and the root system. Leaves were numbered starting at the apex and counting down towards the bottom. One cuvette enclosed a fully expanded mature leaf (leaf # 14–16), which was exposed to ^13^CO_2_ during the labeling period. A second cuvette enclosed the apical bud plus the topmost 3–4 developing leaves. The third cuvette monitored a fully expanded leaf between the ^13^CO_2_ exposed leaf and the plant apex (leaf # 7–10). The last cuvette enclosed the root system. In shoot experiments, two shoots were analyzed in parallel. Each shoot was monitored with two cuvettes: one for a fully expanded mature leaf (leaf # 9–13); and the other one for the apical bud plus 3–4 young developing leaves. One shoot was labeled with ^13^C-glucose and the other shoot was labeled with ^13^CO_2_, as described above. The solution of the latter shoot was continuously sampled for sugar analysis every 2 h during and after ^13^C-labeling. Leaf measurements were related to leaf area, whereas root measurements were related to fresh weight.

Experiments were conducted using long day conditions with the light phase starting at 6:00 CET and ending at 22:00 CET. Measurements were taken throughout the experiment, which lasted until 18:00 CET on the third day. All experiments followed the same sequence: the day before labeling (day 1), plants were adapted to the new environment for 4 h (18:00–22:00 CET) before the lights were switched off (22:00–06:00 CET). In order to ensure steady-state conditions of net CO_2_ assimilation and isoprene emission, the plants were monitored for 4 h after the lights were switched on (06:00–10:00 CET) before labeling was started. Intact plants were labeled with ^13^CO_2_ for 8 h (10:00–18:00 CET). In experiments with shoots, the ^13^C- labeling time was extended by 4 h (10:00–22:00 CET), synchronizing the start of the post-labeling phase with the dark period (22:00–06:00 CET). On day 3, 6 h after the start of the light phase (06:00–12:00 CET), CO_2_-free air was applied by shutting off the CO_2_ supply. After 2 h (12:00–14:00 CET), the lights were switched off for 1 h (14:00–15:00 CET). For the first hour after the lights were switched on again, CO_2_ free air was applied to the shoot experiments, while ^12^CO_2_ containing air was applied to the intact plants (15:00–16:00 CET). Then, ^12^CO_2_ was provided again in all experiments, and the measurements were continued until 18:00 CET of day 3, monitoring post-stress behavior. At the end of the experiment, all leaves were immediately frozen in liquid nitrogen and stored at –80°C for metabolic analysis.

Each cuvette was flushed with 2 L min^-1^ of synthetic, VOC-free and humidified (dew point: +1°C) air containing 385 ppmv CO_2_. The cuvette outlets were connected to electronic valves switching automatically between the cuvettes. The outlet air was directed through a cross junction to a PTR-MS to determine the isotopic composition of the BVOC, and a TDLAS for online analysis of ^12^C^16^O_2_ and ^13^C^16^O_2_. The last port of the cross junction served as a vent for the surplus cuvette air, which was monitored frequently for gas-tightness of the whole system.

### TDLAS measurements of ^12^CO_2_ and ^13^CO_2_

For online quantification of ^12^C^16^O_2_ and ^13^C^16^O_2_ mixing ratios we used a TDLAS (TGA100A, Campbell Scientific, Inc., Logan, UT, USA). Instrumentation and measurement procedures have been described elsewhere [15]. Inlet and outlet air of each cuvette was measured for 105 s each, of which the first 45 s were omitted, before switching to the next cuvette. After sampling two cuvettes, two intervals of 90 s each were added for TDLAS calibration with low (335 ppmv) and high (540 ppmv) CO_2_ concentrations of reference gases (Basi Schöberl, Rastatt, Germany), of which the first 30 s were omitted. Thus, the entire cycle for measuring all four cuvettes was 20 min. Data points shown are 60 s averages within each 20 min cycle.

**Figure 2:**
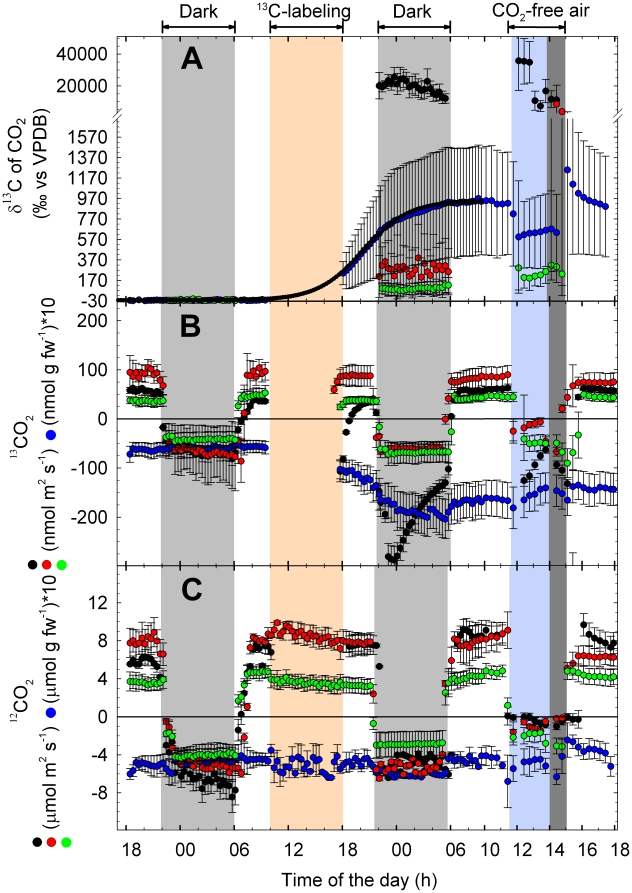
Net assimilation and respiratory CO_2_ emissions of intact poplar plants upon ^13^CO_2_ feeding. (A) Calculated δ^13^C of respired CO_2_ in the absence of net CO_2_ assimilation, assimilation rate of (B) ^13^CO_2_ and (C) ^12^CO_2_ of the mature ^13^CO_2_-fumigated leaf (black symbols), a younger fully expanded leaf (red), the apical bud with enclosed young leaves (green) of intact plants. (B) Release of CO_2_ from root systems immersed in hydroponic solution (blue). Calculated respiratory δ^13^C of roots during the period of ^13^CO_2_ labeling could not be presented (see Materials and Methods), they were replaced instead by sigmoidal fitted data (SigmaPlot v9.0, CA, USA; equation “sigmoid, 3 parameters”; R^2^ = 0.9997) shown in black. The labeling period is shown in orange, the period of stress (absence of CO_2_) by a blue background, and the dark periods are marked grey. Note that the stress period and the last dark period overlap. Data represent the mean of 3 experiments ± s.e.

Net fluxes of ^12^CO_2_ and ^13^CO_2_ were related to projected leaf area and calculated as follows:
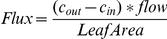



Furthermore, in the absence of net CO_2_ assimilation δ^13^C of plant respiratory CO_2_ was calculated as follows:
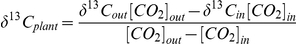
where δ^13^C_out_, δ^13^C_in_, [CO_2_]_out_ and [CO_2_]_in_ are referred to δ^13^C values and CO_2_ concentrations of the cuvette outlet and inlet air, respectively; δ^13^C [‰]  =  (*R_sa_/R_ref_* − 1) × 1000, related to Vienna Pee Dee Belemnite (VPDB), with R_sa_ and R_ref_ as the sample and reference isotope ratios, respectively. No CO_2_ flux measurements for the fumigated mature leaf were possible during the ^13^CO_2_ labeling periods, as ^13^C/^12^C isotope ratio of the ^13^CO_2_ used was far beyond the detection range of the instrument. For experiments with intact plants, a ^13^C memory effect was observed in the TDLAS measurements of the other plant parts during the period of ^13^CO_2_ labeling. Therefore, respiratory δ^13^C of roots could not be precisely determined during fumigation and, thus, data were omitted (see Fig. 2A). Net CO_2_ assimilation rates were calculated according to von Caemmerer & Farquhar [26].

### Measurement of BVOC emissions with PTR-MS

For online monitoring of BVOC a PTR-MS (Ionicon Analytik GmbH, Innsbruck, Austria) was used [27]. Prior to the analysis, cuvette background signals were determined and used for individual background correction of each cuvette. Calibration of the instrument was performed using a mixture of 11 VOC in N_2_ (Apel-Riemer Environmental, Denver, CO, USA) passed through the whole gas exchange system at different concentrations (4–52 ppbv).

Protonated isotopologues masses of emitted BVOC were monitored at m/z (in the following “m” for short) of m33–m34 (methanol), m45–m47 (acetaldehyde), m47–m49 (ethanol), m69–m74 (isoprene), m137–m147 (monoterpenes), m149 and m205 (sesquiterpenes). BVOC produced within the octadecanoid pathway (so called “LOX products”) were monitored at m81 and m99 for hexenal products, and at m83, m101 and m143 for hexenols, hexanal and hexenyl acetate, respectively (for details see [28]).

The percentage of ^13^C incorporated into BVOC was calculated as described in Ghirardo et al. [10]. Protonated ^13^C labeled masses of acetaldehyde at m47 overlapped with the mass of unlabeled protonated ethanol. Thus, analysis of ^13^C incorporation into acetaldehyde was possible only when ethanol did not become significantly labeled (i.e. increase of m48 and m49) or ethanol emission was constant. Under these circumstances, the m47 signal originating from ethanol was taken as background, and the increase of m47 (with corresponding increase of m46 and decrease of m45) was assumed to originate from the incorporation of two ^13^C into acetaldehyde. We checked that the total (^13^C-labeled and unlabeled) acetaldehyde emissions were coherent with acetaldehyde emission during the pre-labeling phase, thus avoiding erroneous interpretation of an increase of the signal at m47 due to a simple increase of unlabeled ethanol emission as ^13^C incorporation into acetaldehyde. Ions with m81 might originate also from fragments of monoterpenes. However, the contribution of monoterpenes to m81 was found marginal due to the very low monoterpene emission. Nevertheless, ions with m81 were considered as hexenal derivatives only when paired with ions at m99.

Total C emitted as BVOC was calculated by multiplying the emission of individual BVOC species with the number of C atoms they contain, and then summing up the values of all compounds. The daily C loss as BVOC was calculated as percentage of total C emitted as BVOC per day divided by the daily net assimilated C, which was determined after subtracting the night respiratory C loss from the assimilated C.

Data for each cuvette were recorded every 20 minutes for 210 s cycle (10 s of flushing plus 200 s of measurements), before switching to the next cuvette. Data of the first 70 s were omitted, and data of the remaining 140 s were aggregated to 20 min means.

### Analysis of ^13^C in bulk plant material, sugars and DMADP, and determination of ^13^C fluxes

An elemental analyzer (Flash EA1110, Thermo Fisher Scientific, Bremen, Germany) coupled to an isotope ratio mass spectrometer (IRMS) (DELTA plus XP, Thermo Fisher Scientific, Bremen, Germany) was used to measure carbon content and ^13/12^C-ratios of the different plant parts. For analysis, 2 mg of dried and homogenized leaf or root material was placed into tin capsules (HeKAtech, Wegberg, Germany). The δ^13^C was measured as described elsewhere [29], [30]. The system was calibrated using three standards (IAEA-CH-3, cellulose; IAEA-CH-6, sucrose; IAEA-CH-7, polyethylene), purchased from the International Atomic Energy Agency (IAEA, Vienna, Austria), and monitored every 11 samples using urea (Sigma Aldrich, Germany) as working standard.

The ^13^C fluxes from the ^13^CO_2_ labeled mature leaf into the nutrient solution containing the unlabeled ^12^Glc (shoot experiments) were calculated as follows:
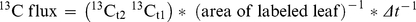
where t_1_ and t_2_ are the two sampling times and *Δt* the difference (in seconds) between the two sampling times. Because the plants continuously took up nutrient solution via the xylem stream, the initial volume was restored before each sampling by adding fresh nutrient solution. Thus, for flux calculation the added carbon at t_2_ (^13^C + ^12^C) was subtracted from the total carbon sampled at t_1_.

The difference (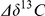) between the δ^13^C of the solution before labeling (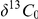) and the δ^13^C determined every 2 h during and after labeling (excluding the night) (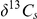) was calculated as follows:



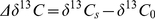
(4)


^13^C/^12^C ratios of the sampled solutions were measured using liquid chromatography coupled to an IRMS (LC-Isolink with DELTA V PLUS, Thermo Fisher Scientific, Bremen, Germany). The IRMS system was calibrated with three reference materials (IAEA C6, sucrose; USGS40, L-glutamic acid; USGS41, L-glutamic acid) purchased from IAEA (Vienna, Austria). Prior to analysis, the aliquots were filtered with a polyvinylidene fluoride (pore size 0.22 µm) filter (Carl Roth GmbH, Karlsruhe, Germany). Due to ^13^C/^12^C fractionation on the filter surface, each individual filter was rinsed with 20 ml of distilled water, and the values were corrected (circa +1‰ vs. VPDB) by means of IAEA reference standards. Drift correction [29] was achieved by interspersing one reference sample (benzoic acid, Sigma Aldrich, Germany) every six samples. The average δ^13^C drift was +0.2‰ within 16 h.

Concentration of sugars (as glucose equivalents) in the solution samples was measured with a phenol-sulfuric acid assay [31] calibrated with six different glucose concentrations (0–20 mM). Positive C flux values indicate downward flux (phloem transport) from the mature leaf to the nutrient solution, whereas negative values indicate the upward transport (to the leaves/apical bud through xylem transport). Leaf DMADP content and relative ^13^C abundance (atom% of total DMADP carbon) was assayed as described elsewhere [10]. Positive C flux values are defined as downward flux (phloem transport) from the mature leaf to the nutrient solution and negative values as the upward transport (to the leaves/apical bud, i.e. xylem transport).

### Statistical Analysis

All labeling experiments were performed in triplicate with the results shown as averages ± s.e. Chemical analyses were performed with three technical replicates. Statistical analyses (t-test, ANOVA) were performed using the Software packages Origin (version 7.0) and Microcal Origin (release 7.0, Microcal Software, Inc., Northampton, MA, USA).

## Results

### ^13^C translocation and allocation in intact plants

In intact plants, ^13^C-labeled photosynthates were translocated from the site of ^13^C fumigation within a few hours, mostly down to the roots, and to a minor extent up to the apex and to the leaves above the ^13^C source leaf, as indicated by the increase in δ^13^C of the respired CO_2_ (Fig. 2A). Root-respired CO_2_ became significantly ^13^C-labeled (δ^13^C value of unlabeled root-respired CO_2_: –24.4 ± 4.3‰) already 1 h after the onset of ^13^CO_2_ fumigation, and followed a sigmoidal increase (R^2^ = 0.9997), which continued during the following dark period until the next day, when the light was switched on again. Based on the length of the stem between the ^13^CO_2_-fumigated leaf and the root system (15–25 cm) and the appearance of the ^13^C-label in root-respired CO_2_ after about 1 h, a phloem transport velocity of 20.3±2.5 cm h^−1^ was estimated.

In the ^13^CO_2_-fumigated leaf, the maximum of respired ^13^CO_2_ appeared approximately 2 h after switching off the lights (Fig. 2B). Application of CO_2_-free air caused a second, strong increase of the ^13^C signal in the CO_2_ released from the labeled leaf, whereas a decrease of ^13^C was observed for root respiration (Fig. 2A), accompanied by a cessation of leaf net CO_2_ assimilation due to the absence of CO_2_ (Fig. 2B, C). Although the ^13^C signal in younger leaves and the apex during dark and CO_2_-free air conditions was not as strong as in the ^13^C-labeled leaf and the roots, the detection of ^13^C provided clear evidence for a significant ^13^C translocation also to these plant parts (Fig. 2A).

**Figure 3:**
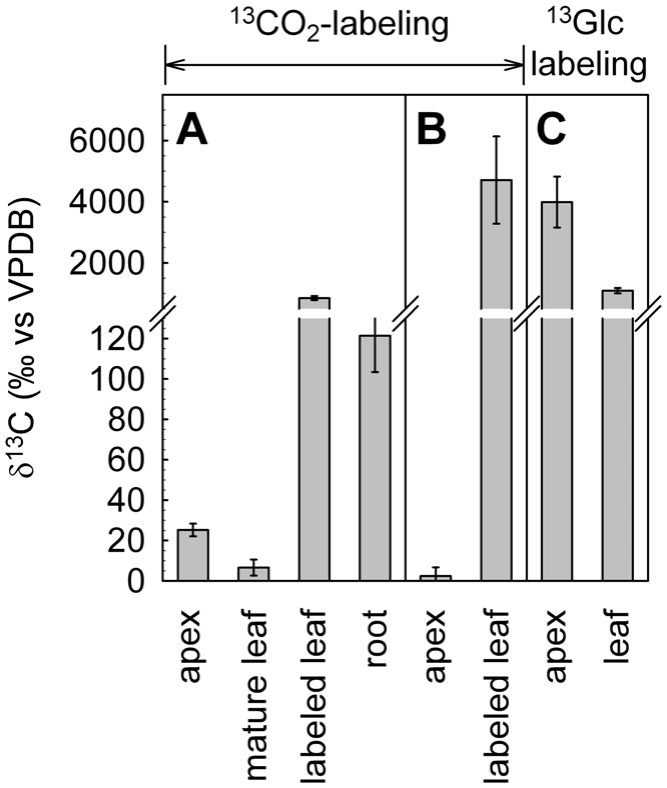
Analysis of δ^13^C in bulk material of poplar. (A) Intact plants labeled with ^13^CO_2_, (B) shoots labeled with ^13^CO_2_, and (C) shoots fed with ^13^Glc. Data represent the mean of 3 experiments ± s.e.

Analyses of the ^13^C content of bulk plant material revealed that ^13^C was, in decreasing order, allocated to and incorporated into (i) the labeled leaf (leaf # 14–16), (ii) the root system, (iii) the apex, and (iv) the mature leaf (leaf # 7–10) (Fig. 3A).

### ^13^C translocation and allocation in shoots

**Figure 4:**
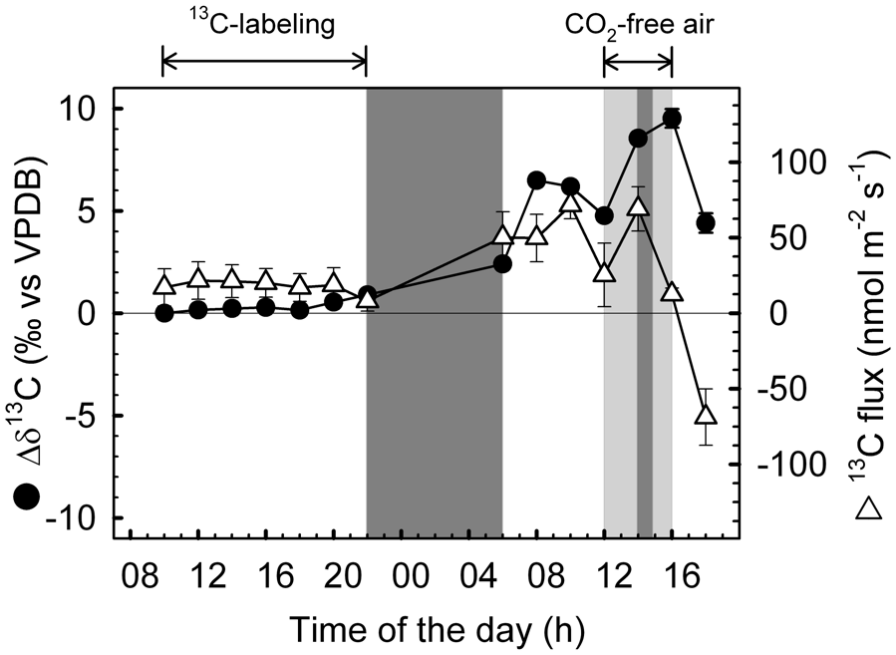
Calculated fluxes of ^13^C-labeled sugars into the nutrient solutions of detached poplar shoots. ^13^C enrichment (black circles) and fluxes (open triangles) of sugars into (positive values) and out of (negative) the nutrient solution of shoots labeled with ^13^CO_2_. The dark phase is indicated with dark grey color. Data represent the mean of 3 experiments ± s.e.

**Figure 5:**
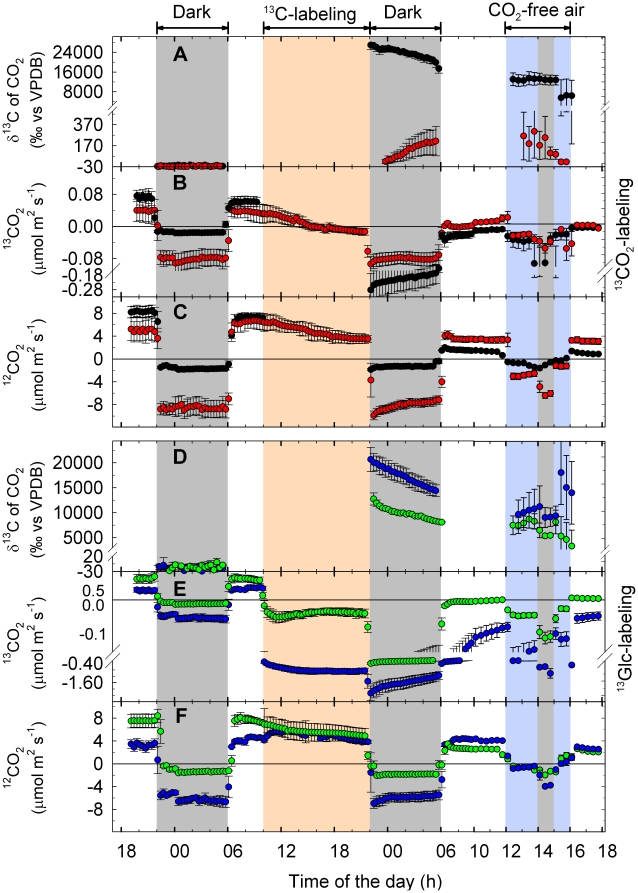
Net assimilation and respiratory CO_2_ emissions of detached poplar shoots upon ^13^CO_2_ and ^13^C-glucose feeding. (A, D) Calculated δ^13^C of respired CO_2_ in the absence of net CO_2_ assimilation, assimilation rate of (B, E) ^13^CO_2_ and (C, F) ^12^CO_2_ in apical buds (red and blue symbols) and mature leaves (black and green symbols) from shoots labeled with (A, B) ^13^CO_2_ or (C, D) ^13^Glc. The labeling period is shown in orange, stress (absence of CO_2_) condition is indicated by a blue background, and dark conditions are marked grey. Note that the stress period and the last dark period overlap. Data represent the mean of 3 experiments ± s.e.

Fumigation of a mature leaf of root-free shoots with ^13^CO_2_ led to a slow ^13^C enrichment of the nutrient solution already during the ^13^CO_2_-labeling period. The ^13^C signal further increased during the following night and the subsequent morning (Fig. 4). Also the ^13^CO_2_ emitted from the apex increased already during the labeling period (Fig. 5B), and the δ^13^C value of respired CO_2_ was higher during the dark phase that increased over time (Fig. 5A).

In shoots labeled with ^13^Glc, ^13^CO_2_ emitted from mature leaves and the apex increased already 20 min after the onset of ^13^C-labeling (Fig. 5E). Respiratory δ^13^C as well as emission of ^13^CO_2_ (Fig. 5D, E) were higher in the apex than in the mature leaf, indicating that the plant apex was the stronger C sink. An increase of ^13^CO_2_ emission was observed in all plant parts during CO_2_-free conditions (Fig. 5B, E).

^13^C label in the ^13^CO_2_ fumigation treatment was highest in the ^13^C-labeled leaf itself, but was also detectable in the apex tissue. When the ^13^C label was supplied as ^13^Glc, ^13^C was dominantly allocated to the young apex leaves (Fig. 3C).

### BVOC emissions, ^13^C incorporation into isoprene and its precursor DMADP

**Figure 6:**
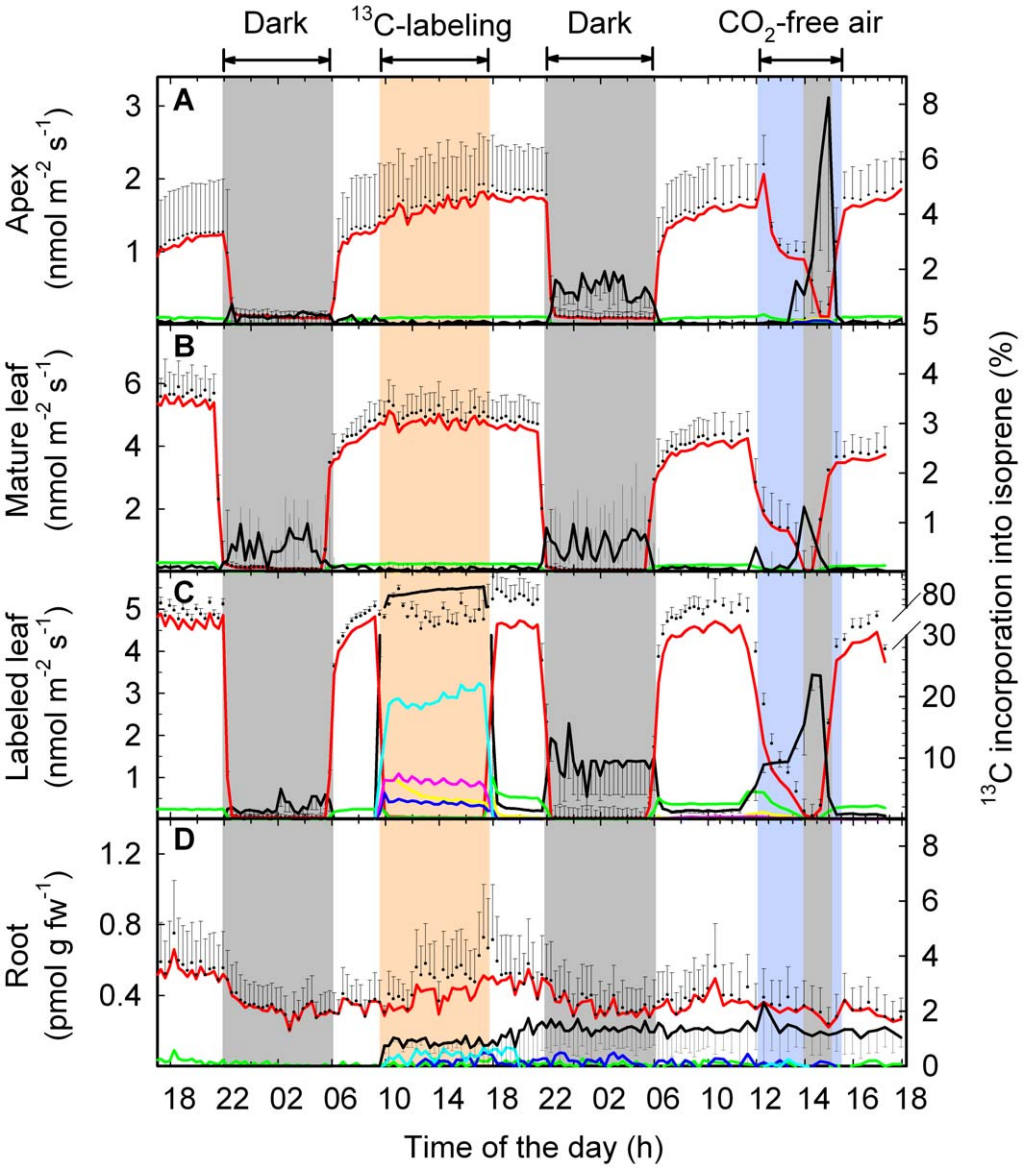
Isoprene emission of intact poplar plants and incorporation of label of upon ^13^CO_2_ feeding. Total isoprene emission (black symbols), its ^13^C incorporation (black line) and isotopic composition (m74, light blue; m73, magenta; m72, dark blue; m71, yellow; m70, green; m69, red) from (A) the apex, (B) a fully expanded leaf, (C) ^13^CO_2_-labeled mature leaf, (D) and the root system of intact plants. The labeling period is shown in orange, stress (absence of CO_2_) is indicated by a blue background, and dark conditions are marked in grey. Details of the experimental phases can be found in the Materials and Methods section. Data represent the mean of 3 experiments ± s.e. (omitted for isotopic composition).

In intact plants, fumigation of an individual leaf with ^13^CO_2_ resulted in a very rapid incorporation of ^13^C into isoprene molecules emitted from the same leaf already during the ^13^CO_2_ fumigation period and, to a lower extent, during the post-labeling period (Fig. 6C). Only a weak incorporation of ^13^C was detectable in root-emitted isoprene. Moreover, we observed no incorporation of ^13^C into isoprene molecules emitted from the apex and younger leaves. In additional experiments, no significant incorporation of ^13^C into isoprene in leaves at position +1, +4, +7, +8, +11, +14, +16 higher than the ^13^C-labeled leaf could be detected (data not shown). Nevertheless, when CO_2_-free atmosphere was applied on day 3, isoprene molecules emitted from the formerly ^13^CO_2_-fumigated leaf became rapidly enriched in ^13^C, and after approximately 1 h also isoprene molecules emitted from the apex showed an increased incorporation of ^13^C (Fig. 6C and A, respectively). In contrast, isoprene originating from younger unlabeled leaves was not significantly labeled (Fig. 6B). Immediately after restoring normal ^12^CO_2_ and light conditions, the ^13^C signature of isoprene returned to its initial pattern (Fig. 6).

**Figure 7:**
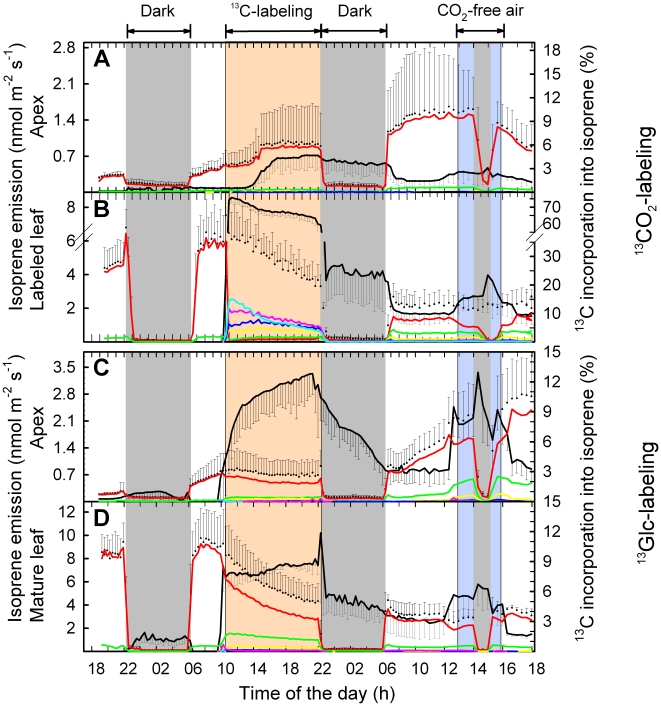
Isoprene emission of detached poplar shoots and ^13^C incorporation upon ^13^CO_2_ and ^13^C-glucose feeding. Total isoprene emission (black symbols), its ^13^C incorporation (black line) and isotopic composition (m74, light blue; m73, magenta; m72, dark blue; m71, yellow; m70, green; m69, red) in (A, C) apical buds and (B, D) mature leaves from shoots labeled with (A, B) ^13^CO_2_ or (C, D) ^13^Glc. The labeling period is shown in orange, stress (absence of CO_2_) is indicated by a blue background, and dark conditions are marked in grey. Details of the experimental phases can be found in the Materials and Methods section. Data represent the mean of three experiments ± s.e. (omitted for isotopic composition).

In shoots, continuous fumigation with ^13^CO_2_ for 12 h or ^13^Glc application for 12 h resulted in a partial incorporation of ^13^C into isoprene emitted from the apex of 4.4±1.6% and 10.8±0.8%, respectively (Fig. 7A, C). In leaves of root-free shoots, ^13^Glc contributed 7.4±1.1% to the total isoprene carbon release after 1 h, and 9.3±0.6% after 12 h of ^13^C-labeling (Fig. 7D).

**Figure 8:**
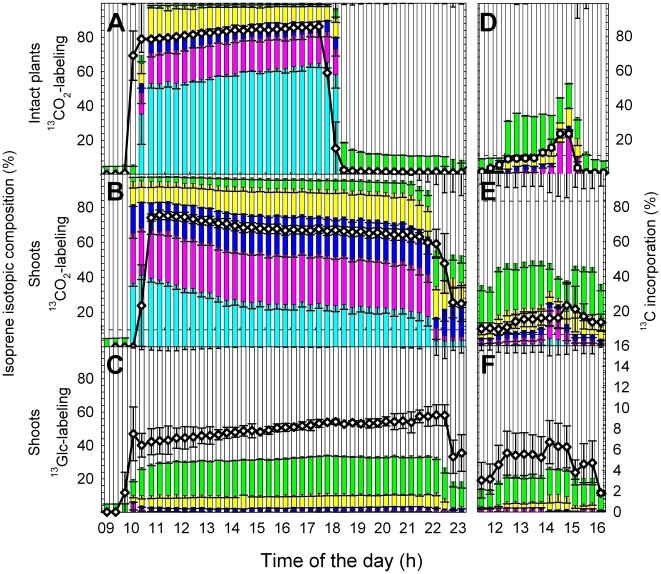
Isotopic composition of isoprene molecules and the corresponding ^13^C-incorporation during ^13^C-labeling of intact poplar plants and detached shoots. Pattern of isoprene isotopologues (m74, light blue; m73, magenta; m72, dark blue; m71, yellow; m70, green; m69, white) and ^13^C-incorporation (open circles) in (A) intact plants, (B) shoots labeled with ^13^CO_2_, and (C) shoots labeled with ^13^Glc. Panels (D), (E), and (F) show the isotopic composition of isoprene emitted by the same plants during the period with CO_2_-free air. Details of the experimental phases can be found in the Materials and Methods section. Data represent the mean of 3 experiments ± s.e.

Feeding intact poplar plants and detached shoots with the two main C sources for isoprene biosynthesis resulted in different fractions of ^13^C-labeled isoprene molecules (Fig. 6, 7). After 1 h of labeling with^ 13^CO_2_, fumigated leaves of intact plants and detached shoots incorporated 76–78% ^13^C into isoprene (Fig. 6C, 7B). The remaining 22–24% originated from C sources other than recently fixed atmospheric CO_2_. Over 8 h of ^13^CO_2_-labeling, the ^13^C incorporation into isoprene molecules increased continuously up to 86.3±0.2% (Fig. 6C), indicating that also alternative C sources became partially ^13^C-labeled. Isoprene molecules became gradually enriched in m74 (Fig. 8). This indicated that a fraction of one or both precursors of chloroplastic isoprene, pyruvate (PYR) and glyceraldehyde 3-phosphate (GAP) originated from plant/leaf-internal C pools with slow turnover. As an indication of the depletion of unlabeled carbon pools, the isoprene fraction with m71 decreased over the 8 h labeling period from 18±2.3% down to 8.2±0.4%, inversely proportionally to m74, which increased from 54±3.1% to 65±3.1% (Fig. 8). Therefore, we estimate that about 10% of carbon was derived from “older” C not originating from recent ^13^CO_2_ fixation. Subjecting the previously ^13^CO_2_-labeled leaves to CO_2_-free atmosphere and darkness led to a significant (P<0.01) increase of isoprene molecules with m70–m73 (containing one to four ^13^C atoms) (Fig. 8D, E). In contrast, in ^13^Glc-fed shoots only isoprene molecules containing one or two ^13^C-atoms (m70 and m71) could be detected (Fig. 8F).

In experiments with detached shoots, total isoprene emission as well as net CO_2_ assimilation rates decreased by 20–50% over the measurement period (Fig. 5B–C, 5E–F, 7B, and 7D). The initial incorporation of 75.8±2.7% ^13^C into isoprene decreased over time down to 63.3±4.0% in shoots fumigated with ^13^CO_2_ and fed with ^12^Glc (Fig. 7B), whereas in the inverse experiment the fraction of ^13^C-labeled isoprene molecules increased over time from 6.5±1.1% up to 9.4±1.0% during ^13^Glc feeding and ^12^CO_2_ fumigation (Fig. 7C).

**Figure 9:**
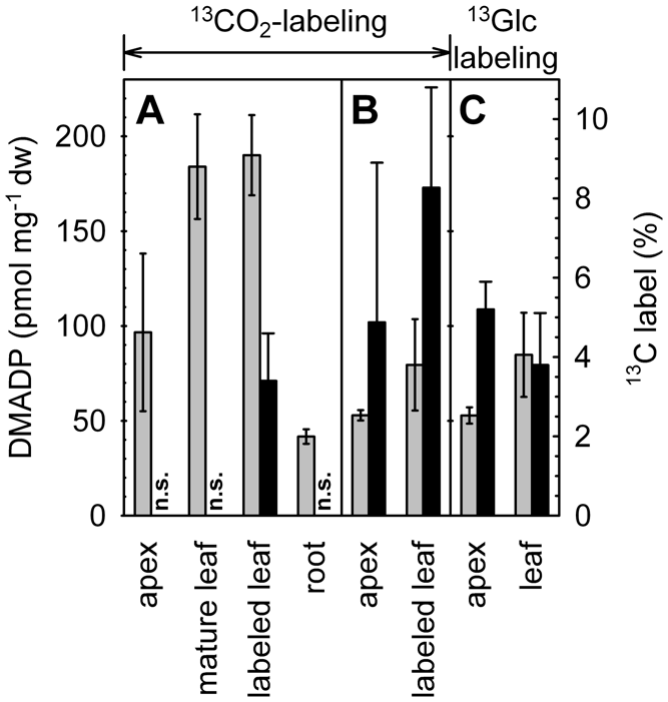
Concentration of the isoprene precursor DMADP and incorporation of ^13^C in DMADP molecules in different plant parts of intact poplar plants and detached shoots. DMADP content (grey bars) and ^13^C-incorporation into DMADP (black bars) in (A) apex, mature leaf, labeled leaf and root of intact plants labeled with ^13^CO_2_, in (B) apex and leaves of shoots labeled with ^13^CO_2_, and in (C) labeled with ^13^Glc. Leaf DMADP content and relative ^13^C-abundance (% of ^13^C in total DMADP carbon) was assayed as described by Ghirardo *et al.* [10]. Data represent the mean of 3 experiments ± s.d. (n.s.: no significant ^13^C-enrichment).

Our dataset documented an age-dependent accumulation pattern of the isoprene precursor DMADP (Fig. 9), concomitant with an age-dependent emission rate of isoprene (Fig. 6, 10E). Young developing leaves (leaves # 1–4) reached emission levels of only 15–30% compared to emission rates of mature leaves (leaves # 7–10; Fig. 6A, B). The isoprene emission slightly decreased with leaf age or lower position in the plant canopy (leaves # 14–16; Fig 6C). The leaf position (i.e. age) effect was also visible over the duration of the experiment, with an increase of isoprene emission in young leaves and decrease in mature leaves (Fig. 6A, B, C). In experiments with detached shoots, lower DMADP pools were accompanied by lower isoprene emission rates (Fig. 7 and 9). We also observed a very low, but still detectable emission of isoprene from the root system (Fig. 6C), which might have resulted from chemical conversion of DMADP into isoprene, since no isoprene synthase activity was detectable in root protein extracts (data not shown).

In contrast to the rest of the plant, the DMADP pool in the labeled leaves of intact plants was still remarkably enriched in ^13^C at the time of harvest (3.4±1.2% ^13^C; Fig. 9A), whereas isoprene emitted from these leaves was less enriched in ^13^C at the end of the experiment (0.8±0.1%). In all detached shoots, the DMADP pools of both the apex and the mature leaves were found to be ^13^C-labeled (Fig. 9B, C). The total DMADP content was similar and highest (187±25 pmol mg^−1^ dw) in unlabeled and ^13^C-labeled mature leaves of intact plants, but was significantly lower in other parts of intact plants and in detached shoots (Fig. 9).

**Figure 10:**
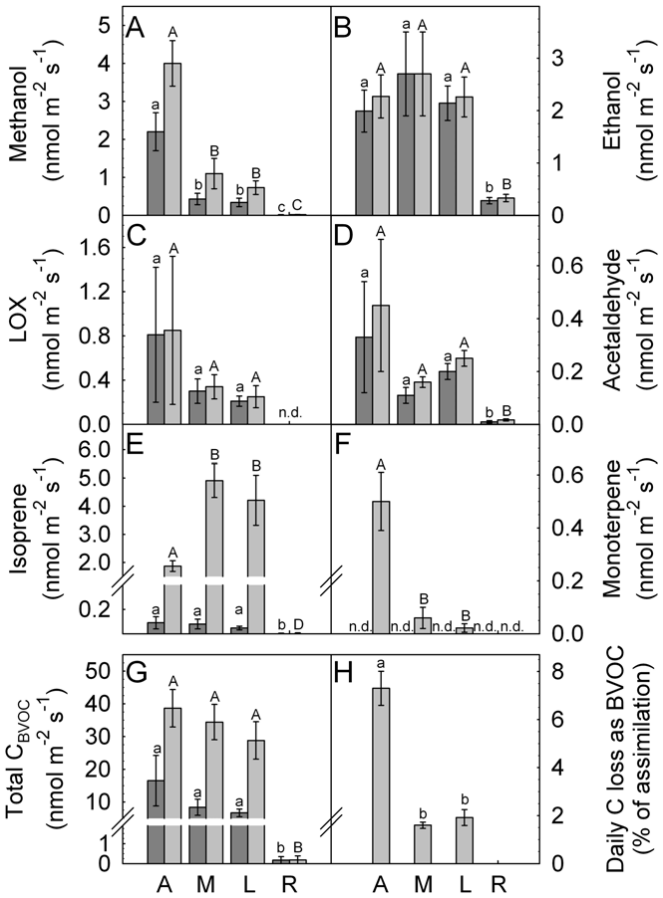
Tissue specific emission rates of BVOC from poplar plants. Summary of (A) methanol, (B) ethanol, (C) LOX products, (D) acetaldehyde, (E) isoprene, (F) monoterpene, (G) total quantity of C loss as BVOC, average emissions during night (dark grey bars) and light (light grey bars) from (A) apex, (M) mature leaves younger than (L) labeled leaves, (R) root system in intact plants. The daily percentage of C loss (H) as BVOC related to daily net assimilation was calculated during day 2 (excluded for R). Data represent the mean of 3 experiments ± s.e. (n.d.  =  not detectable). Statistical significant differences (t-test with p<0.05) of emissions between A, M, L and R are given with different minuscule or capital letters for dark or light emissions, respectively.

Analysis of plant BVOC emissions with PTR-MS allowed quantification of all volatile compounds emitted by poplar. Isoprene was the dominant – though not the only – BVOC emitted in mature leaves (emissions of individual BVOC are summarized in Fig. 10). Young developing leaves emitted significantly higher amounts of methanol and monoterpenes and lower amounts of isoprene as compared to mature leaves (Fig. 10A, F, E). Emission of sesquiterpenes, a group of semi-volatile terpenes, was not detectable in all measurements (data not shown).

Overall, we calculated a daily C loss as BVOC relative to daily net CO_2_ assimilation of 7.3±0.7% for developing leaves and 1.9±0.3% for mature leaves (Fig. 10H). In young developing leaves, other non-isoprene BVOC accounted to approximately 50% of total C loss as BVOC during the light phase, whereas their contribution in mature leaves was only 27% (Table 1). During darkness, when light-dependent emission of isoprene was negligible (4–5%, Table 1), we calculated that total C loss as BVOC was still 43% and 24% of the daily C loss in young leaves and mature leaves, respectively. Thus, non-isoprene BVOC (e.g. methanol) became the major species of plant C loss during the night.

**Table 1:** Contribution of C loss (%) as isoprene in different plant's parts relative to total C loss as BVOC during night and light emissions in experiments with intact plants.

Plant part	Night	Light
Apex	4.4±2.1	51±18
Mature leaf*	4.8±1.4	73±7
Labeled leaf	3.6±0.7	73±2
Roots	2.1±0.6	2.6±0.7

Mean of three individual experiments ± s.e.

(*  =  mature leaf younger than labeled leaf).

Significant ^13^C-labeling was found in methanol emitted from both the apices (8.9±2.7%) and mature leaves (7.4±0.1%) of shoots labeled with ^13^Glc, whereas no significant incorporation of ^13^C could be detected in shoots fumigated with ^13^CO_2_. The immediate start of the dark period after ^13^C labeling revealed an incorporation of ^13^C into acetaldehyde during the post-illumination peak [28] of 27±7%. In all other non-isoprene BVOC no significant ^13^C label was detected.

## Discussion

### ^13^C translocation and allocation in intact plants

Our data reveal the dynamics of C translocation from mature leaves of intact and unstressed grey poplar plants. The export of photoassimilates was directed primarily downward to the root system via the phloem. This agrees with a former ^14^C-labeling study with *Populus deltoides* [32], where the ^14^C label was primarily found in the roots after 72 h. However, the present analysis showed that this allocation of C in the roots was a very fast process that occurred already in a few hours, and because of the real-time measurements, the analysis allowed assessing the phloem velocity, information which is scarcely reported in the literature. The estimated phloem velocity of 20.3±2.5 cm h^−1^ in our experiment can explain the link of photosynthesis and ecosystem respiration observed in other recent studies. In a coniferous forest, soil respiration was linked to net CO_2_ assimilation with a time lag of 1–4 d [16]. A time lag of 4–5 d for the C transfer of recently fixed assimilates down to the root stock was reported for a deciduous forest [17]. Högberg *et al.* [19] and Subke *et al.* [20] observed lag times of 2–4 d between ^13^CO_2_-labeling of whole pine trees and the appearance of ^13^C in soil CO_2_ efflux. Furthermore, the present data show that the downward transport of C from mature leaves to the root system of poplar was highest during the night.

We used hydroponics with plants immersed in a sterile nutrient solution to avoid bacterial respiration. Therefore, we are quite certain that the magnitude of the CO_2_ release reflects root respiration. Because half of the soil CO_2_ efflux might originate from recent photoassimilates [18], the C loss of the plant as root exudates is another important aspect in constraining ecosystem C fluxes and C budgets.

Freshly fixed C was partly converted to compounds for storage and transport, very likely starch and sucrose, the main storage and transport forms in plants [33]. Accumulation of starch granula in chloroplasts and amyloplasts varies diurnally during net CO_2_ assimilation, whereas starch degradation to sucrose happens in the absence of net photosynthesis (e.g., at night) in order to sustain plant growth and metabolism [22], [34], [35]. For the ^13^CO_2_-labeled leaf, the observed time pattern of ^13^CO_2_ release might be well explained by an intermediate storage of ^13^C as starch during ^13^C-labeling that served as carbon and energy source during the following dark period or during increased energy demand (stress) [36]. Starch grain synthesis occurs in layers, of which those that were accumulated last are remobilized first [37]. Thus, we speculate that the “onionskin” structure might explain the observed dynamics of ^13^CO_2_ emission during the dark period after labeling. Since the saplings were exposed to unlabeled CO_2_ for 4 h between the end of the ^13^C-labeling phase and the following night, leading to synthesis of unlabeled starch, the ^13^C content of respired CO_2_ gradually increased after switching off the light. Once solely ^13^C-labeled starch started to be remobilized, the ^13^C content of the CO_2_ emitted increased to its maximum, approximately after 2 h of darkness, i.e. half of the time of assimilation of unlabeled CO_2_ between the two phases. This time response agrees well with the diurnal carbon balance of starch accumulation during the light phase and starch mobilization during nighttime, where the diurnally accumulated starch pool is linearly consumed during the night until the starch is almost completely utilized at the end of the night [36]. Thus, probably due to the selected photoperiod of 16/8 h light/dark cycle, the ^13^C peak in CO_2_ 2 h after onset of darkness occurred exactly after half of the time of the unlabeled phase before darkening. When the ^13^C-labeled starch layers of the grains were consumed, unlabeled starch layers were mobilized, consequently leading to a decrease in the ^13^CO_2_ signal of respiration.

In poplar, sucrose is the major sugar form of the phloem sap, whereas glucose and fructose are the dominating sugars transported in the xylem [22]. A significant proportion of C was translocated via the phloem from mature ^13^C-labeled source leaves down to the root system where most of the ^13^C was stored and used only partially for root catabolism at night. This observation proves clearly the function of roots as carbohydrate reserves [38].

### ^13^C translocation and allocation in shoots

The experiments with root-free poplar shoots confirmed that assimilated ^13^C was translocated downward via the phloem, reflected by the increased ^13^C content of the sugars found in the nutrient solution of the shoots over time. Contrary to intact plants, the deprivation of the root system short-circuited the process of C storage in the roots, and labeled ^13^C could be transported directly to the apical part of the plant. The ^13^C-labeled sugars entering the nutrient solution could immediately enter the xylem stream again and be transported upward, mainly to the C sink tissue of the apex.

In poplar shoots fed with ^13^Glc via the transpiration stream, ^13^Glc was allocated to all leaves, but mainly to the strong C sink tissue of the apex in the absence of net photosynthesis. In contrast, in intact poplar saplings, the ^13^C flux from mature, ^13^C-labeled leaves was directed mainly downward.

In the same way as for intact plants, the diurnal dynamics of ^13^C-labeled starch synthesis in shoots and its successive degradation during the dark phase well explain also the ^13^CO_2_ release pattern during our experiments. Immediate mobilization of labeled starch caused an initial maximum release of ^13^CO_2_, which exponentially decreased over time, since in the case of detached shoots the ^13^C-labeling period had been extended until the onset of darkness.

### Incorporation of ^13^C into isoprene and its alternative C sources

Potential alternative, xylem-dependent C sources of isoprene biosynthesis [13] were not directly influenced by recently fixed C of a single mature leaf. After feeding ^13^CO_2_ to leaves, no appreciable ^13^C signal appeared in isoprene molecules emitted from adjacent upper leaves. Although we observed a slight increase of ^13^CO_2_ release from the upper younger leaves, no such ^13^C incorporation was found in isoprene molecules emitted from the same leaves. Because the whole poplar sapling contributed directly or indirectly to source-sink reallocation, the portion of C allocated from one single mature labeled leaf [32] might have been too small to be detectable in isoprene molecules with PTR-MS.

The xylem-transported sugars contributed only as a minor C source to isoprene formation. Nevertheless, the fraction found in the present study was significantly higher than the 4% (in absence of stomatal closure) reported in a previous study with poplar [13]. This might be explained either by the higher concentration of ^13^Glc applied in the present study (here 10 mM, compared to 5 mM in [13]), or by the different way of application (here via shoots, in [13] via the petioles). However, we have to mention that in both studies total isoprene emission and net CO_2_ assimilation rates dropped significantly, probably due to a wounding response because of cutting. In the present study, this decline amounted to 20–50% over the measurement period, whereas a larger decrease of 80% occurred during the experiment with cut petioles [13]. We cannot exclude that this effect might also have had consequences for the magnitude of the alternative xylem-dependent C source.

Isoprene was not fully labeled even after prolonged exposure of the leaves to ^13^CO_2_. However, ^13^C incorporation increased constantly during 8 h of ^13^C-labeling. Together with our findings from both ^13^CO_2_- and ^13^Glc-labeling experiments and the related isotopologue composition of isoprene, we have indications that alternative C sources of isoprene formation are not only based on xylem-transported sugars. A significant fraction is also related to metabolic pools with longer turnover times, for example starch degradation and re-fixation of mitochondrial CO_2_. In the present study the origin of C emitted as isoprene was (i) 76–78% from recent photosynthesis, (ii) about 8–11% from photosynthates with slower turnover rates, and (iii) about 9–10% from xylem-transported sugars. Overall, we were able to account for 93–99% of the C sources of isoprene formation. The remaining 1–7% might originate from chloroplastic degradation of starch occurring simultaneously to starch biosynthesis, as isoprene was still 1–2% labeled the day after ^13^CO_2_-labeling of leaves. Another possibility might be due to re-fixation of mitochondrial C [13] or simply part of the experimental error.

The observed isoprene emission potential in different plant parts perfectly matches the developmental activation pattern of the promoter of isoprene synthase (*PcISPS*) [24]. However, we observed only a marginal emission of isoprene from the root stock, albeit the promoter of the *PcISPS* gene was activated [24]. Although the amount of DMADP found in the roots was lower compared to other plant parts, the root DMADP pool was five orders of magnitude higher than the associated isoprene emission, compared to the DMADP to isoprene ratio in leaves. Thus, isoprene emission from roots can be likely explained as a consequence of natural, enzyme-independent DMADP decay to isoprene at physiological pH [10], [39], [40] of cytosolic DMADP pool.

Isoprene biosynthesis is located in chloroplasts and closely related to photosynthesis. The cytosolic DMADP pool sustains the mevalonate pathway and accounts for circa 75–90% of the total (chloroplastic plus cytosolic) pool, whereas the chloroplastic DMADP pool is rapidly depleted during darkness [41], [42]. The isotopic composition of isoprene molecules reflected the isotopic composition of chloroplastic DMADP [10]. After a short time of ^13^CO_2_-labeling, the incorporation into isoprene was higher than in total DMADP due to the cytosolic DMADP pool. At the end of the experiment, the day after ^13^C-labeling, we found higher incorporation of ^13^C in the total DMADP pool than in emitted isoprene molecules. Thus, we have to assume (i) that to a certain extent chloroplastic ^13^C-labeled intermediates were transported into the cytosol, and (ii) that the chloroplastic DMADP pool of the MEP pathway undergoes a faster turnover than the cytosolic DMADP pool of the mevalonate pathway.

With our setup it is possible to measure most of significant volatile carbon molecules, (except volatiles with lower proton affinity than H_2_O, [27]), CO_2_ and BVOC of grey poplar plants. In addition to isoprene, we observed monoterpene emission from young developing tissue as earlier reported for young leaves of *Populus tremula* [43] and *Populus euroamericana* [44]. However, the biological reason(s) for the decrease of monoterpene and increase of isoprene emission with leaf aging is still unclear. The strong emission of methanol in young tissue [45] is attributed to the activity of pectin methylesterases [46], which among other functions, demethoxylate pectin during cell expansion in all types of plant tissue. The metabolic origin of acetaldehyde, e.g. appearing for a short period after switching off the lights, and ethanol emitted by trees is still a matter of debate [7]. However, the relatively high emission of ethanol and acetaldehyde at all leaf levels of poplar might result from somehow anoxic conditions in the hydroponic solutions since flooding has been shown to stimulate ethanol production which, after transport to the leaves, is oxidized to acetaldehyde [47].

In our study, we successfully traced online the dynamics of C fluxes and we determined C allocation within the poplar plants. We showed that for the biosynthesis of isoprene, part of the “alternative C sources” originated from a transport of C via the root system. Overall, the present dataset proves that the combination of isotope-specific analysis (TDLAS and PTR-MS), in conjunction with tissue-specific ^13^C-labeling, allows online quantification of the plants' respiratory CO_2_ and BVOC emissions, which in turn can be used to infer the translocation of C in poplar. Together with metabolic analysis, it paves the way for comprehensively analyzing metabolic shifts in plants under various environmental conditions.
